# Patterns of Food Parenting Practices and Children’s Intake of Energy-Dense Snack Foods

**DOI:** 10.3390/nu7064093

**Published:** 2015-05-27

**Authors:** Dorus W. M. Gevers, Stef P. J. Kremers, Nanne K. de Vries, Patricia van Assema

**Affiliations:** 1 Department of Health Promotion, NUTRIM School for Nutrition and Translational Research in Metabolism, Maastricht University Medical Centre, P.O. Box 616, Maastricht 6200, The Netherlands; E-Mails: s.kremers@maastrichtuniversity.nl (S.P.J.K.); n.devries@maastrichtuniversity.nl (N.K.d.V.); p.vanassema@maastrichtuniversity.nl (P.v.A.); 2 Department of Health Promotion, CAPHRI School for Public Health and Primary Care, Maastricht University Medical Centre, P.O. Box 616, Maastricht 6200, The Netherlands

**Keywords:** energy-dense snack foods, children, food parenting practices, cluster analysis, clustering, patterns, obesity

## Abstract

Most previous studies of parental influences on children’s diets included just a single or a few types of food parenting practices, while parents actually employ multiple types of practices. Our objective was to investigate the clustering of parents regarding food parenting practices and to characterize the clusters in terms of background characteristics and children’s intake of energy-dense snack foods. A sample of Dutch parents of children aged 4–12 was recruited by a research agency to fill out an online questionnaire. A hierarchical cluster analysis (*n* = 888) was performed, followed by k-means clustering. ANOVAs, ANCOVAs and chi-square tests were used to investigate associations between cluster membership, parental and child background characteristics, as well as children’s intake of energy-dense snack foods. Four distinct patterns were discovered: “high covert control and rewarding”, “low covert control and non-rewarding”, “high involvement and supportive” and “low involvement and indulgent”. The “high involvement and supportive” cluster was found to be most favorable in terms of children’s intake. Several background factors characterized cluster membership. This study expands the current knowledge about parental influences on children’s diets. Interventions should focus on increasing parental involvement in food parenting.

## 1. Introduction

Childhood obesity has become a problem in many countries [1], including the Netherlands, where overweight prevalence rates have increased from about 5% of boys and 7% of girls in 1980 to about 13% and 15%, respectively, in 2009 [2]. These figures are alarming, as being overweight during childhood is associated with several health problems, including cardiovascular, metabolic and psychosocial problems [3]. Additionally, an increasing number of studies have indicated that being overweight in childhood persists during later life [4], so encouraging healthy energy balance-related behaviors in young children is important. One of the behavioral aims for interventions could be to reduce the consumption of energy-dense nutrient-poor foods, which include non-core foods, such as potato chips, chocolate and cookies. Although more research is needed to disentangle the association between the consumption of these foods and children’s weight status, there is ample evidence indicating that intakes of such foods have increased substantially in U.S. children in recent decades, as well as some evidence that the frequency of snacking is positively related to total energy intake [5]. The observed U.S. trends could also be indicative of other Western countries. In the national food consumption survey 2007–2010 in the Netherlands (NFCS), children were found to have an average of about three energy-dense snack food (EDSF) occasions a day, yielding more than 1500 kJ [6]. Less than 10% of the children researched met dietary guidelines regarding energy intake from non-core foods, illustrating the need for programs addressing this behavior.

Preventive actions might focus on home and family factors. Parents have been shown to be important environmental agents, as children are dependent on them with regard to food intake [7]. This dependency concerns a variety of food parenting practices (FPPs), such as bringing foods into the home and storing them, setting rules about food intake or modeling healthy food habits [8].

Traditionally, research has focused on relationships between individual FPPs and the child’s diet (or aspects thereof). This approach implies that a few individual practices or constructs are treated as independent predictors, while exploring this relationship, for instance, by examining them by means of bivariate correlations or single regression models (e.g., [9,10,11,12,13,14]). The dynamics of parenting practices have scarcely been examined. We found two studies from the United States that examined the interrelatedness of parenting practices [15,16]. One of them investigated vegetable parenting practices, which clustered according to three theorized dimensions (*i.e.*, responsiveness, structure and control) [16], while the other [15] found three clusters of parents: one cluster of parents reporting low use of FPPs, one cluster of parents reporting high use of FPPs and the third a “non-directive” cluster (*i.e.*, “enhanced availability and teachable moments and less firm discipline practices”). In a Dutch sample, evidence was found for interrelatedness between diet- and activity-related parenting practices [17]. These three studies all had different objectives (confirming *vs*. exploring the co-occurrence of parenting practices) and included different types of practices (vegetable, food and both physical activity and food), but all contribute to the evidence base on food parenting, thereby increasing the potential of future interventions. The present study was intended to extend this evidence base by examining the interrelatedness of a set of FPPs among a sample of Dutch parents.

Studying the co-occurrence of a wide range of FPPs has a great advantage over the traditional “isolated” approach. Parents do not employ single practices, but actually employ several of them, which might have synergistic [17] or opposing effects. This might be revealed by examining patterns, which is not possible using isolated observations. Moreover, demographic characteristics might be used to profile clusters of parents, which would create opportunities to target parents belonging to particular clusters. For instance, parental educational level has emerged as an important correlate for all clusters discovered across the food and physical activity domain [17]. The clusters found by O’Connor *et al*. [15] were associated with ethnicity, parental age and child age, all pointing toward potential target groups.

The aim of this study was to assess the co-occurrence of FPPs. We formulated three research questions. (1) Which clusters of parents can be distinguished, based on their food parenting practice pattern? (2) Which parental and child background factors characterize cluster membership? (3) Does the intake of EDSFs among children differ according to their parents’ cluster membership?

## 2. Methods

### 2.1. Study Design and Participants

This study used baseline data from an ongoing prospective study. Parents were recruited by a research agency (Flycatcher Internet Research), which meets quality standards for social science research and access panels, as indicated by the ISO20252 and ISO26362 standards [18]. Their panel consists of about 16,000 Dutch people aged 12 years and older who receive credit points in exchange for their participation in online questionnaire research. On average, panel members are invited to participate in such research eight times a year. For the present study, panel members were invited if they had at least one child aged 4–12; this applied to 1985 persons. Data were retrieved from two questionnaires (together constituting the baseline measurement of the larger study) sent off in October and November 2014, respectively. Participants received 500 credit points for completion of both, which equals 5.56 Euros. If parents had more than one child aged 4–12, they were asked in the first survey to answer all questions about the child whose birthday came first, counting from the moment of invitation, to limit potential confounding. To remind parents in the second survey of the child of interest, we used information from the first (*i.e.*, children’s age, gender and first name). Four to seven days after both invitations, non-responders received a reminder by e-mail. This study was exempt from review by a research ethics committee, as it does not fall within the scope of the Dutch Medical Research Involving Human Subjects Act [19].

### 2.2. Measures

#### 2.2.1. Parent and Child Background Information

Respondents indicated their work status (*i.e.*, employed *versus* unemployed) and reported their postal code, enabling us to calculate a factor score indicating their socio-economic position (SEP; −4 (low)–4 (high). This score has been developed by the Netherlands Institute for Social Research and is based on income, educational level and occupation [20]. Height and weight were self-reported, enabling us to calculate body mass index (BMI). The respondents’ age, gender, educational level and ethnicity were known to the research agency. Children’s age, gender, height and weight were reported by the respondents. Height and weight data were compared with a 1997 Dutch reference population [21] to calculate the child’s BMI-z score.

#### 2.2.2. Food Parenting Practices

FPPs were examined using the Comprehensive Snack Parenting Questionnaire (CSPQ) [22]). This 21-item questionnaire was developed for the purpose of mapping 21 distinct food parenting behaviors related to snack food intake. Items showed acceptable test-retest reliability statistics (intra-class correlations ranging from 0.41–0.70; exact agreement scores ranging from 60%–70% for items with intra class correlations ≤0.40) [22]). The CSPQ assesses practices categorized using a model of general parenting [23]: Responsiveness, including encouragement, rewarding, discussing, providing feedback, involving, educating, healthy modeling and unhealthy modeling avoidance; structure, including the availability of healthy foods, accessibility of healthy foods, visibility of healthy foods, limited availability of unhealthy foods, limited accessibility of unhealthy foods, structure and meal routines; behavioral control, including permissiveness, rules and monitoring; and finally, psychological control, including instrumental feeding, emotional feeding and pressure to eat. For example, parents were asked to rate the following statements: “I give [child’s first name] EDSFs to make [him/her] feel better”; “I teach [child’s first name] things about food”; and “I consciously refrain from eating EDSFs when [child’s first name] is around”, with answer categories ranging from 1, strongly disagree, to 5, strongly agree.

#### 2.2.3. Intake of Energy-Dense Snack Foods

Thirteen questions assessed the child’s number of energy-dense food occasions per week (with relevant examples of what constitutes energy-dense snack foods provided). Parents were asked to respond to the question: “Thinking about the last month, how many days a week did your child normally consume: (1) potato chips, (2) candy (e.g., wine gums, lollipops), (3) chocolates, (4) candy bars, (5) dried fruit biscuits, (6) small or medium sized cookies such as Dutch windmill cookies, (7) large cookies such as chocolate chip cookies, (8) pie or pastry (e.g., pie, apple turnover), (9) savory snacks and peanuts (e.g., nuts, popcorn), and (10) gum throughout the day and (11) ice creams, (12) deep fried snacks or (13) gingerbread between meals.” Answering categories ranged from never to 7 days a week. These scores were summed to create a variable indicating the number of energy-dense food occasions per week. The food items were taken from a validated Dutch food frequency questionnaire assessing children’s energy intake [24,25].

### 2.3. Statistical Analysis

A logistic regression analysis was performed to determine differences between responders and non-responders with regard to the parent’s age, gender, educational level and ethnicity.

Before running cluster analyses, we standardized food parenting practice scores, recoded univariate outliers (*i.e.*, values > 3 SD distance from the mean) and eliminated multivariate outliers (*i.e.*, cases with large Mahalanobis distances [26]). Additionally, data were checked for multicollinearity, applying a cut-off value of 0.80; patterns of FPPs were examined in a two-step procedure [27]. The first step involved the use of a hierarchical cluster analysis using Ward’s method and squared Euclidean distances. To determine the optimal number of clusters, we created a scree plot of the agglomeration coefficients and visually inspected the dendrogram. The second step of the procedure involved the performance of a non-hierarchical cluster analysis (*i.e.*, k-means) with varying cluster solutions to discover which solution most meaningfully distinguished parents in terms of their FPPs. The stability of the final cluster solution was assessed using a cross-validation procedure [28]. To this end, data were randomly split into two subsamples (Samples A and B), and the full two-step cluster analysis was replicated in both. In addition, the resulting obtained cluster centers of Subsample A were saved and used to classify the participants from Sample B into a second set of clusters. By calculating Cohen’s kappa, we assessed the similarity of the two Sample B solutions. We considered kappa >0.6 to be acceptable [29]. The final clusters were interpreted and labeled using the four factors [23] categorizing individual parenting practices: Responsiveness, structure, behavioral control and psychological control. ANOVAs (for continuous variables) and chi-square tests (for categorical variables) were used to investigate associations between cluster membership and parental and child background characteristics, including parental age, gender, BMI, ethnicity, SEP, working status and educational level, as well as the child’s age, gender and BMI-z score. If the overall *F*-test or chi-square test resulted in a *p*-value < 0.05, *post hoc* comparisons were performed using Tukey HSD (following *F*-tests) or Bonferroni (following chi-square tests).

ANCOVAs followed by Bonferroni *post hoc* test were used to assess differences between clusters in terms of a child’s energy-dense food intake, while correcting for background characteristics found to be significantly related to the clusters. IBM SPSS Statistics 20 was used for all analyses.

## 3. Results

### 3.1. Sample

In total, 939 parents (response 47.3%) filled out both questionnaires completely, 888 of which were eligible (*i.e.*, having a child aged 4–12 years old) and had complete responses of sufficient quality (*i.e.*, no straight-lining responses as assessed by the research agency and no multivariate outliers) and were thus entered into the data analysis. Parents with low (OR = 1.85; 95% CI = 1.39–2.46; *p*-value < 0.01) and intermediate (OR = 1.40; 95% CI = 1.2–1.7; *p*-value < 0.01) educational levels were less likely to fill out the questionnaire than parents with a high educational level. There were no differences between responders and non-responders with regard to their age, gender and ethnicity.

Parents had a mean age of 40.6 (SD = 5.8; range 25–64). More than half of the sample was female (65.2%), and about one tenth of the sample had a low educational level. Parents with an intermediate educational (45.3%) and high educational level (43.6%) were equally represented. Most of the parents were of Dutch ethnicity (91.0%) and were employed (79.3%). The parents had an average BMI of 25.2 (SD = 4.2), with 0.6% being classified as underweight (*i.e.*, <18.5 kg/m^2^), 54.2% as normal weight (*i.e.*, 18.5–24.9 kg/m^2^) and 45.2% as overweight (*i.e.*, ≥25 kg/m^2^). The mean SEP score was 0.1 (SD = 1.2). Compared to the Dutch population, female parents and parents with medium and high educational levels (about 41% and 40%, in the Dutch population, respectively) and being of Dutch origin (about 70% in the Dutch population) were overrepresented in the sample [30]. With regard to BMI, the sample resembled self-reported national figures, but objectively measured height and weight indicate a slightly higher overweight rate in the Dutch population as a whole [31]. Children of participating parents (49.7% female) had a mean age of 7.9 (SD = 2.6) and a mean BMI-z score of 0.19 (SD = 1.4). According to Barlow’s cut-off points [32], approximately 12% of the children were underweight, 73% had a healthy weight and 15% were overweight or obese. The number of overweight children corresponds to the national population [2]. On average, children had 12.4 (SD = 5.9) energy-dense snack food occasions per week.

### 3.2. Identification of Clusters

**Figure 1:**
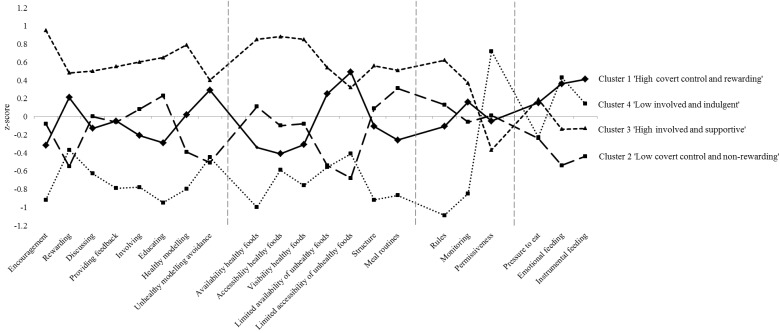
Graphical view of the four-cluster solution based on mean z-scores for all food parenting practices (*n* = 888); Higher scores indicate more frequent use of the FPP; Each of the successive graphical areas represents a distinct category of food parenting practices, *i.e.*, responsiveness, structure, behavioral control and psychological control; Cluster 1 “high covert control and rewarding”: solid line; Cluster 2 “low covert control and non-rewarding”: long dashed line; Cluster 3 “high involvement and supportive”: short dashed line; Cluster 4 “low involvement and indulgent”: dotted line.

Before running cluster analyses, univariate outliers were replaced by the mean score plus three standard deviations (1.6% of all responses on FPPs), and multivariate outliers (19 cases) were eliminated from the further analyses. No multicollinearity was observed between the included FPPs (all Pearson’s correlations ≤0.63). Ward’s method indicated that a four-cluster solution gave the best fit, in view of the change in agglomeration coefficients. After considering this outcome and the dendrogram, we conducted k-means cluster analyses using 4-, 5-, 6- and 7-cluster solutions, from which a four-cluster solution was derived (Table 1; Figure 1). After replicating the full two-step clustering approach in two subsamples, we obtained a Cohen’s kappa of 0.97, indicating substantial stability of the cluster solution.

The first cluster (*n* = 273; 30.7%) was labeled “high covert control and rewarding” and was characterized by relatively high scores on avoidance of unhealthy modeling, high scores on limited availability and accessibility of unhealthy foods and high use of instrumental and emotional feeding. Cluster 2 (*n* = 227; 25.6%) consisted of parents with low scores on healthy modeling and avoidance of unhealthy modeling, low scores on limited availability and accessibility of unhealthy foods and low use of practices with a rewarding component (*i.e.*, rewarding, instrumental and emotional feeding) and was called “low covert control and non-rewarding”. The third cluster (*n* = 247; 27.8%) was named “high involvement and supportive” because members of this cluster had high scores for the use of parenting practices from the responsiveness, structure and behavioral control categories, but low scores for permissiveness. Parents in Cluster 4 (“low involvement and indulgent”; *n* = 141; 15.9%) had relatively low scores on all types of FPPs, except for permissiveness.

### 3.3. Characterizing Cluster Membership

Cluster membership was characterized by parental age, gender, BMI and SEP, as well as child’s age and BMI-z score (Table 2). Parents from Cluster 1 (*i.e*., “high covert control and rewarding”) appeared to be older than those from Cluster 4 (*i.e.*, “low involvement and indulgent”). Cluster 2 (*i.e.*, “low covert control and non-rewarding”) included more woman than Cluster 4. Parents in Cluster 4 were found to have higher BMI scores than parents from Clusters 1 and 2 and tended to have a higher SEP than Cluster 2 parents. Children of parents from Clusters 2, 3 (*i.e.*, “high involvement and supportive”) and 4 were older than their peers from Cluster 1, while those from Clusters 2 and 4 were older than those from Cluster 3. Parents from Clusters 1 and 4 had children with higher BMI-z scores than parents from Cluster 2, and those from Cluster 4 had also higher BMI-z scores than those from Cluster 3.

**Table 1:** Four-cluster solution: Mean z-scores for all food parenting practices (*n* = 888).

		Cluster 1 High Covert Control and Rewarding *n* = 273 (30.7%)		Cluster 2 Low Covert Control and Non-rewarding *n* = 227 (25.6%)		Cluster 3 High Involvement and Supportive *n* = 247 (27.8%)		Cluster 4 Low Involvement and Indulgent *n* = 141 (15.9%)
**Responsiveness**								
Encouragement		−0.32		−0.08		**0.95**		**−0.92**
Rewarding		**0.21**		**−0.55**		**0.48**		−0.37
Discussing		−0.13		0.00		**0.50**		**−0.63**
Providing feedback		−0.05		−0.06		**0.55**		**−0.79**
Involving		−0.21		0.08		**0.60**		**−0.78**
Educating		−0.29		0.23		**0.65**		**−0.95**
Healthy modelling		0.02		**−0.39**		**0.79**		**−0.80**
Unhealthy modelling avoidance		**0.29**		**−0.51**		**0.40**		−0.45
**Structure**								
Availability of healthy foods		−0.34		0.11		**0.85**		**−1.00**
Accessibility of healthy foods		−0.41		−0.10		**0.88**		**−0.59**
Visibility of healthy foods		−0.31		−0.08		**0.85**		**−0.76**
Limited availability of unhealthy foods		**0.25**		**−0.54**		**0.54**		**−0.56**
Limited accessibility of unhealthy foods		**0.49**		**−0.68**		0.32		**−0.41**
Structure		−0.11		0.09		**0.56**		**−0.92**
Meal routines		−0.26		0.31		**0.51**		**−0.87**
**Behavioral Control**								
Rules		−0.11		0.13		**0.62**		**−1.09**
Monitoring		0.16		−0.06		0.37		**−0.85**
Permissiveness		−0.05		0.01		**−0.37**		**0.72**
**Psychological Control**								
Pressure to eat		0.15		−0.24		0.19		−0.23
Emotional feeding		**0.36**		**−0.54**		−0.14		0.43
Instrumental feeding		**0.41**		**−0.44**		−0.13		0.14

Note: Higher scores indicate more frequent use of the food parenting practice (FPP); Bold numbers indicate scores used to label the clusters; Higher scores indicate more frequent use of the FPP; Each of the successive graphical areas represents a distinct category of food parenting practices, *i.e.*, responsiveness, structure, behavioral control and psychological control; Cluster 1 “high covert control and rewarding”: solid line; Cluster 2 “low covert control and non-rewarding”: long dashed line; Cluster 3 “high involvement and supportive”: short dashed line; Cluster 4 “low involvement and indulgent”: dotted line.

**Table 2:** Cluster membership: Means (SD) and proportions according to background characteristics and the child’s number of snack occasions per week.

	Total Sample	Cluster 1 High Covert Control and Rewarding	Cluster 2 Low Covert Control and Non-rewarding	Cluster 3 High Involvement and Supportive	Cluster 4 Low Involvement and Indulgent	*p*-value Overall *F* or Chi-square Test	Cluster Differences
**Parent characteristics**							
Age (years), mean SD	40.6 (5.8)	40.0 (5.5%)	41.2 (5.4%)	40.2 (6.3%)	41.6 (6.0)	0.01 ^a^	4 > 1 ^c^
Gender (female) (%)	65.2%	64.8%	71.4%	65.2%	56.0%	0.03 ^b^	2 > 4 ^d^
BMI (kg/m^2^), mean SD ^1^	25.2 (4.2)	24.9 (4.1)	25.0 (4.0)	25.3 (4.1)	26.2 (4.3)	0.01 ^a^	4 > 1,2 ^c^
Ethnicity (Dutch ethnicity) (%)	91.0%	90.1%	94.3%	89.9%	89.4%	NS ^b^	NA
SEP (factor score), mean SD ^2^	0.1 (1.2)	0.2 (1.1)	0.0 (1.3)	0.1 (1.3)	0.4 (1.0)	0.03 ^a^	4 > 2 ^c^
Work status (in employment) (%)	79.3%	76.2%	79.3%	79.8%	84.4%	NS ^b^	NA
**Educational level**						NS ^b^	NA
Low (%)	11.1%	12.1%	12.3%	7.7%	13.5%		
Medium (%)	45.3%	46.2%	43.6%	43.3%	49.6%		
High (%)	43.6%	41.8%	44.1%	49.0%	36.9%		
**Child characteristics**							
Age (years), mean SD	7.9 (2.6)	7.1 (2.6)	8.5 (2.4)	7.7 (2.6)	8.5 (2.6)	< 0.01 ^a^	2, 3, 4 > 1; 2 > 3 ; 4 > 3 ^c^
Gender (female) (%)	49.7%	49.5%	50.2%	54.3%	41.1%	NS ^b^	NA
BMI-z, mean (SD)	−0.2 (1.4)	−0.1 (1.4)	–0.4 (1.2)	−0.3 (1.4)	0.1 (1.5)	< 0.01 ^a^	1, 4 > 2; 4 > 3 ^c^
EDSFs (occasions per week), mean SD ^3^	12.4 (5.9)	11.9 (5.5)	13.2 (5.9)	11.2 (5.7)	13.7 (6.3)	< 0.01 ^a, e^	2, 4 > 3 ^d, e^

Note: Total sample: *n* = 888 except where otherwise specified; Cluster 1 (*n* = 273), Cluster 2 (*n* = 227), Cluster 3 (*n* = 247), Cluster 4 (*n* = 141); ^1^ *n* = 871, ^2^ higher scores indicate higher socio-economic position (SEP) (range −4–4) (*n* = 881), ^3^ uncorrected means (SD) are presented, ANCOVAs: *n* = 864; ^a^ *p*-value overall *F*-test, ^b^ *p*-value chi-square test, ^c^ Tukey HSD *post hoc* test, ^d^ Bonferroni *post hoc* test, ^e^ parental age, gender, BMI, SEP and child age and BMI-z included as covariates; NS: not significant; NA: not applicable.

**Figure 2:**
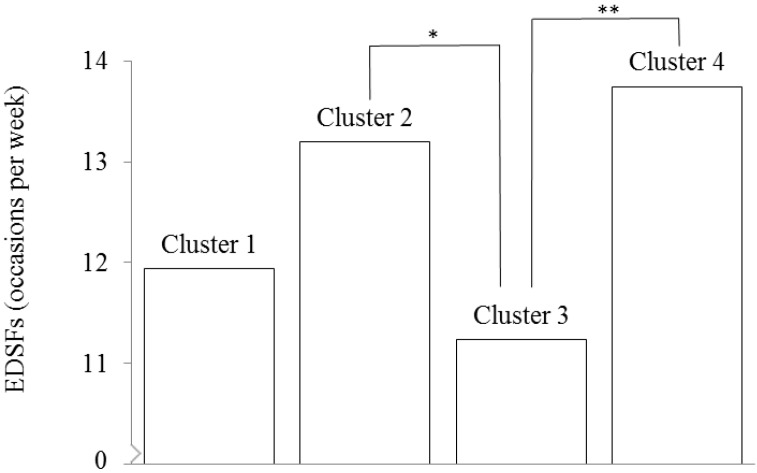
Mean energy-dense snack food (EDSF) occasions per week according to cluster membership; * *p* < 0.05, ** *p* < 0.05, uncorrected means (SD) are presented (*n* = 888), ANCOVAs: *n* = 864, Cluster 1 = “high covert control and rewarding”; Cluster 2 = “low covert control and non-rewarding”; Cluster 3 = “high involvement and supportive”; Cluster 4 = “low involvement and indulgent”.

The number of EDSF occasions per week was the lowest among children from Cluster 3 (mean = 11.2, SD = 5.7) and differed significantly from the intake among children from Clusters 2 (mean = 13.2, SD = 5.9) and 4 (mean = 13.7, SD = 6.3; Table 2, Figure 2). The mean number of EDSF occasions was 11.9 (SD = 5.5) among children from Cluster 1 parents.

## 4. Discussion

Most previous studies of parental influences on children’s diets applied a narrow and isolated focus on a single or a few types of food parenting practices (FPPs), while parents actually employ multiple types of practices. The combination of the practices likely influences child behavior, as opposed to independent influences of separate practices [33]. We therefore used a clustering approach to examine the interrelatedness of FPPs and investigated how these patterns relate to parental and child characteristics, including children’s intake of energy-dense snack foods (EDSFs).

A two-step cluster analysis revealed four distinct patterns, of which the “high involvement and supportive cluster” was found to be the most favorable in terms of children’s intake of EDSFs. Parents in this cluster tended to be involved in communicating about food, created a clear and supportive home food environment and are relatively strict regarding the application of snacking rules. Children of parents from the “low covert control and non-rewarding” and “low involvement and indulgent” clusters had significantly higher intakes, in spite of several relatively favorable scores on individual parenting practices (e.g., pressure to eat). About 40 percent of the parents we studied were characterized by these two patterns. The discovery of a low involvement food parenting pattern is in line with the results of O’Connor *et al.* [15]. Finally, a “high covert control and rewarding” pattern was identified in about 30% of the parents, consisting of parents that applied relatively many covert control practices with respect to unhealthy foods (e.g., keeping few snacks in the house), but tended to use snacks to reward or soothe their child. The label of this and the “low covert control and low rewarding” pattern were derived from Brown and Ogden’s work [34], who defined covert control as “controlling a child’s food intake in a way that cannot be detected by the child” and includes parental behaviors, such as avoiding bringing unhealthy foods into the house and trying not to eat unhealthy foods when their children are around [34]. 

Previously established relationships between FPPs and children’s snack food intake could be used to illustrate the dependence of FPPs on their wider context. For instance, availability and accessibility of snack foods have been found to be undesirable [35], as well as instrumental and emotional feeding [14,36]. The present study shows that low availability and accessibility of unhealthy foods in the home environment with instrumental and emotional feeding (*i.e.*, Cluster 1) were more favorable than the opposite pattern (*i.e.*, Cluster 2). Thus, FPPs from the psychological control category might be less detrimental in the context of high covert control (*i.e.*, limiting unhealthy foods in the home and avoiding unhealthy modeling). In addition, previous studies on isolated FPPs may have produced spurious results. To illustrate, studies on restrictive rules among Dutch parents [37,38,39] could have overstated its impact, as the measure of rules might have served simply as an indicator of a highly involved and supportive pattern, rather than as a measure of the effect of strict rules *per se*.

Our results regarding the four patterns and our attempt to profile them provide some directions for the design of interventions aimed at preventing childhood obesity. We found cluster differences based on parental age, gender, BMI and SEP and child’s age and BMI. Developers of family interventions might consider tailoring to these factors to fit the needs and desires of potential users or targeting specific subgroups at high risk for low involvement in food parenting. We performed a sensitivity analysis by exploring FPP patterns in three different strata (based on child’s age) and discovered that the low involvement pattern was more pronounced among parents of older children. Therefore, parents may need assistance in preventing a shift towards this pattern, and we suggest directly addressing parents who are not highly involved with food parenting. Further work is planned to predict the use of FPPs based on parental characteristics, such as behavior-specific cognitions (e.g., perceived behavioral control, attitude and intention), personality and demographics.

Although the cluster solution appeared to be stable within the sample, the technique has an explorative nature, so further research is needed to validate the findings and to demonstrate the value and potential impact of FPP clusters in longitudinal designs. Replication of the present study is possible, as we used a standardized measure of food parenting. It should be noted that few parents in our sample had a low educational level and few were immigrants, which reduces the representativeness of the sample. Future work using the same approach should pay particular attention to the representativeness of the sample to establish external validity. The self-report measures were probably biased towards socially-desirable scores, which is a well-known phenomenon in parenting research [40]. The children’s EDSF intake scores may have been biased, in particular by recall bias, considering the low frequency of occasions compared to figures derived from 24-hour recall data in the Dutch NFCS [6]. It is unknown if the impact (*i.e.*, size and direction) of this bias differed between the clusters.

## 5. Conclusions

By examining the co-occurrence of a set of FPPs, our study expands the knowledge base on parenting practices used to regulate children’s food intake behaviors. Specifically, the study took account of the interdependence of practices. Parents were distinguished based on their FPP patterns, using four clusters. Differences between clusters were found in children’s EDSF intake, with the “high involvement and supportive” cluster found to be the most favorable and the “low involvement” cluster the least favorable. Additional studies are needed to verify the existence of the patterns that we identified.
